# Supplementary material for: New disordered anyon phase of doped graphene zigzag nanoribbon
Source: Sci Rep. 2022 Aug 25;12:14551. doi: 10.1038/s41598-022-18731-6 (PMC9411593; doi:10.1038/s41598-022-18731-6)
Supplement: Supplementary file 1 — Supplementary Information. [file 41598_2022_18731_MOESM1_ESM.pdf]

Supplementary material: New disordered anyon phase of doped graphene zigzag nanoribbon

Young Heon Kim,[1] Hye Jeong Lee,[1] Hyun-Yong Lee,[2,3,4] and S.-R. Eric Yang[1,*]

[1]*Department of Physics, Korea University, Seoul 02855, Korea*

[2]*Department of Applied Physics, Graduate School, Korea University, Sejong 30019, Korea*

[3]*Division of Display and Semiconductor Physics, Korea University, Sejong 30019, Korea*

[4]*Interdisciplinary Program in E·ICT-Culture-Sports Convergence, Korea University, Sejong 30019, Korea*

(Dated: July 20, 2022)

I. MATRIX PRODUCT STATES

A large class of quantum many-body wavefunctions, Ψ, can be efficiently factorized into a product of tensors as follows:

$$|\psi\rangle = \sum_{\{s_i\}} \Psi_{\cdots s_{i-1}s_i s_{i+1}\cdots} \, \big|\cdots s_{i-1}s_i s_{i+1}\cdots\big\rangle$$
$$= \sum_{\{s_i\}} \mathrm{tTr}[\cdots A_{i-1}^{s_{i-1}} A_i^{s_i} A_{i+1}^{s_{i+1}} \cdots] \big|\cdots s_{i-1}s_i s_{i+1}\cdots\big\rangle, \tag{S1}$$

where $A_i^{s_i}$ stands for a tensor at site i, $\cdots A_{i-1}^{s_{i-1}} A_i^{s_i} A_{i+1}^{s_{i+1}} \cdots$ indicates the product of tensors forming a network, depending on how tensors are connected, and $\mathrm{tTr}[\cdots]$ stands for the tensor trace or contraction of all connected indices in the network. Here the index s_i denotes a local state at site i, e.g., $|s_i\rangle = |\uparrow\rangle, |\downarrow\rangle$ for a spin-half fermion system or $|s_i\rangle = |0\rangle, |\uparrow\rangle, |\downarrow\rangle, |\uparrow\downarrow\rangle$ for a spin-full fermion system. Particularly, the so-called MPS, which is a chain-like product of rank-3 tensors, may represent quantum states for one-dimensional and quasi-one-dimensional systems accurately. Specifically, the many-body wave function in the MPS representation is written as follows:

$$\Psi_{\cdots s_{i-1}s_i s_{i+1}\cdots} = \sum_{\{l_i\},\{r_i\}} \cdots \delta_{r_{i-2}l_{i-1}} \, [A_{i-1}^{s_{i-1}}]_{l_{i-1},r_{i-1}} \, \delta_{r_{i-1}l_i} \, [A_i^{s_i}]_{l_i,r_i} \, \delta_{r_i l_{i+1}} \, [A_{i+1}^{s_{i+1}}]_{l_{i+1},r_{i+1}} \, \delta_{r_{i+1}l_{i+2}} \cdots, \tag{S2}$$

where $[A_i^{s_i}]_{l_i,r_i}$ is a rank-3 tensor with two virtual indices l_i and r_i (say left and right, respectively), which are traced out, s_i is the physical index, and δ_{ij} stands for the Kronecker delta or the identity matrix. Note that the Kronecker delta contracts the right and left indices of tensor at i and $i+1$, respectively. Hence, the element of the wavefunction for a given set of $\{s_i\}$ is identical to the product of *matrices* $\{A_i^{s_i}\}$, i.e., $\Psi_{\cdots s_{i-1}s_i s_{i+1}} = \cdots A_{i-1}^{s_{i-1}} A_i^{s_i} A_{i+1}^{s_{i+1}} \cdots$. It is also convenient to introduce a graphical representation for the tensor and its network. A tensor is depicted by an object with open legs denoting its indices. For instance, the tensor $A_i^{s_i}$ can be illustrated as follows:

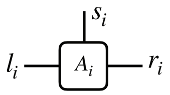

$$= [A_i^{s_i}]_{l_i r_i}, \tag{S3}$$

where the vertical open leg s_i denotes the physics index and the horizontal ones l_i and r_i stand for the virtual indices. Furthermore, a contraction of two indices, particularly one left and one right horizontal indices, occurs by connecting the legs (or indices) as shown below:

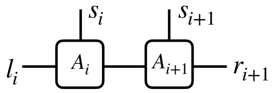

$$= [A_i^{s_i}]_{l_i r_i} \, \delta_{r_i l_{i+1}} \, [A_{i+1}^{s_{i+1}}]_{l_{i+1} r_{i+1}}. \tag{S4}$$

Thus, the total wavefunction in the MPS representation is illustrated as follows:

$$\Psi_{\cdots s_{i-1}s_i s_{i+1}\cdots} =$$

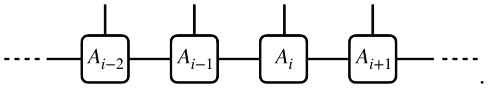

(S5)

Here, the size of the matrix $A_i^{s_i}$, which is referred to as the bond dimension χ, determines the expressibility of the MPS. In other words, the accuracy of the MPS can be systematically enhanced by increasing the value of χ. We define the MPS on the zigzag nanoribbon in a so-called snake pattern, as illustrated in Fig. S1. This definition allows us to consider the graphene strip with long zigzag edges.

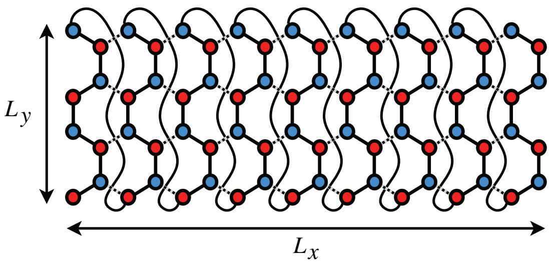

Fig. S1. Schematic figure of the snake pattern of the matrix product state defined on the zigzag nanoribbon lattice.

Similarly, a large class of Hamiltonians can be represented exactly as a product of matrices or as the matrix product operator (MPO) as follows:

$$\begin{aligned}\hat{H} &= \sum_{\{s_i\},\{s_i'\}} H^{\cdots s_{i-1}' s_i' s_{i+1}' \cdots}_{\cdots s_{i-1} s_i s_{i+1} \cdots} \left| \cdots s_{i-1}' s_i' s_{i+1}' \cdots \right\rangle \left\langle \cdots s_{i-1} s_i s_{i+1} \cdots \right| \\ &= \sum_{\{s_i\},\{s_i'\}} \mathrm{tTr}[\cdots W^{s_{i-1}'}_{i-1,s_{i-1}} W^{s_i'}_{i,s_i} W^{s_{i+1}'}_{i+1,s_{i+1}} \cdots] \left| \cdots s_i' \cdots \right\rangle \left\langle \cdots s_i \cdots \right|, \end{aligned} \tag{S6}$$

where $W^{s_i'}_{i,s_i}$ is a rank-4 tensor with its graphical representation given as

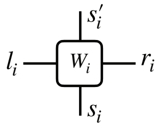

$$= [W^{s_i'}_{i,s_i}]_{l_i r_i} \quad . \tag{S7}$$

Then, the Hamiltonian is represented in the graphical representation as follows:

$$H^{\cdots s_{i-1}' s_i' s_{i+1}' \cdots}_{\cdots s_{i-1} s_i s_{i+1} \cdots} =$$

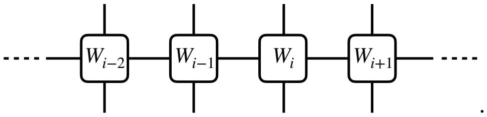

$$. \tag{S8}$$

For example, the Hamiltonian of the one-dimensional Anderson–Hubbard model is produced with the tensor $W^{s'}_{i,s}$ as follows:

$$[W^{s'}_{i,s}]_{lr} = \begin{bmatrix} (I_i)_{ss'} & 0 & 0 & 0 & 0 & 0 \\ (c^\dagger_{i\uparrow})_{ss'} & 0 & 0 & 0 & 0 & 0 \\ (c^\dagger_{i\downarrow})_{ss'} & 0 & 0 & 0 & 0 & 0 \\ (c_{i\uparrow})_{ss'} & 0 & 0 & 0 & 0 & 0 \\ (c_{i\downarrow})_{ss'} & 0 & 0 & 0 & 0 & 0 \\ (U n_{i\uparrow} n_{i\downarrow} + v_i(n_{i\uparrow} + n_{i\downarrow}))_{ss'} & t(c_{i\uparrow})_{ss'} & t(c_{i\downarrow})_{ss'} & -t(c^\dagger_{i\uparrow})_{ss'} & -t(c^\dagger_{i\downarrow})_{ss'} & (I_i)_{ss'} \end{bmatrix}_{lr}, \tag{S9}$$

where I_i is the trivial operator acting on site i, $c_i^{(\dagger)}$ is the annihilation (creation) operator, v_i is the Anderson random potential, and U is the Hubbard interaction. One can easily generalize the above $W-$tensor and MPO into the proper quantities for the model of the quasi-one-dimensional honeycomb lattice.

The total energy of a given wavefunction is given as

$$E = \frac{\langle \Psi | H | \Psi \rangle}{\langle \Psi | \Psi \rangle} = \frac{\sum_{\{s_i\},\{s_i'\}} (\Psi_{\cdots s_i' \cdots})^* H^{\cdots s_i' \cdots}_{\cdots s_i \cdots} (\Psi_{\cdots s_i \cdots})}{\sum_{\{s_i\}} (\Psi_{\cdots s_i \cdots})^* (\Psi_{\cdots s_i \cdots})}, \tag{S10}$$

and this total energy is graphically recast as

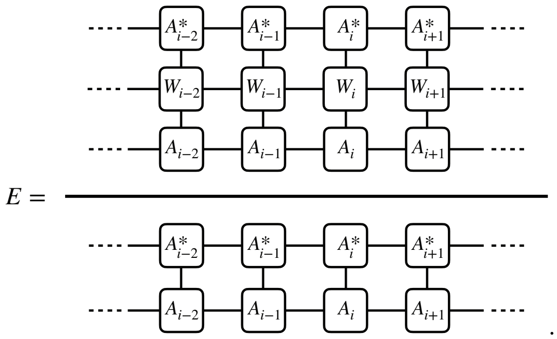

(S11)

Thus, to measure the total energy E, one should contract two different tensor networks in the denominator and numerator in the above equation. The contraction of tensor networks can be efficiently conducted by choosing the order of the contraction properly[1]. With the above expression, one can apply a variational principle to optimize tensors $\{A_i\}$ by minimizing the energy, e.g., $\partial E/\partial A_i^* = 0$, Thereby leading to an eigenvalue problem to update the tensor A_i (see Ref.[1] for more details).

A. Density Matrix Renormalization Group: Clean limit

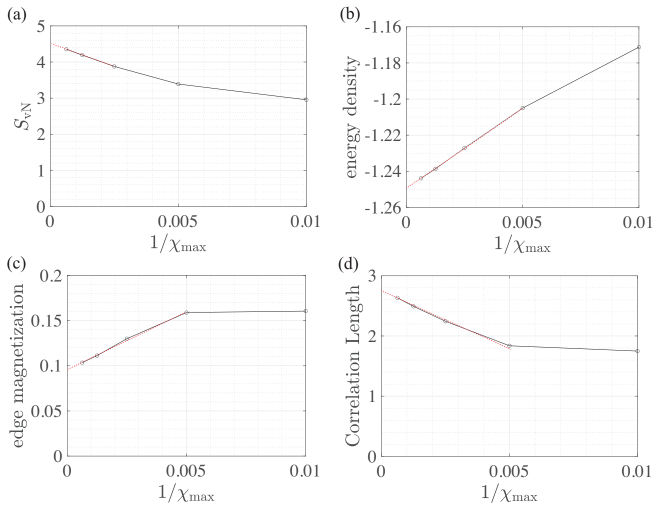

Fig. S2. The bond dimension scaling of entanglement entropy (a), energy density (b), edge magnetization ($\hbar = 1$) (c), and correlation length (d). Here $U/t = 1$ is used. The energy unit is t and the length unit is $\sqrt{3}a$, where a is the carbon-carbon distance.

As discussed in the previous section, the bond dimension of the MPS determines the amount of the MPS. The number of MPS should be carefully chosen to appropriately represent the ground state. Specifically, the MPS is optimal for representing quantum states that satisfy the area law of the entanglement, i.e., its entanglement entropy scales as the size of the boundary between two subsystems. By contrast, the entanglement entropy of a gapless ground state diverges logarithmically with the subsystem size. Thus, we first check whether the ground state of our Hamiltonian is gapped or gapless. Then, we determine how large the bond dimension should be to appropriately represent the ground state. To this end, we perform the infinite-size variant of the DMRG [1] and see how the energy density, total magnetization, entanglement entropy scale and correlation length as a function of the maximum bond dimension $\chi_{\rm max}$. The result is presented in Fig. S2. It is certain that the entanglement entropy does not diverge but converges to a finite value as $\chi_{\rm max} \to \infty$ [see red dashed line in Fig. S2(a)], which implies that the ground state is gapped or satisfies the area law of the entanglement. Also, based on the scaling result of the energy density and magnetization, as shown in Figs. S2(a) and S2(b), we conclude that keeping 1600 states, i.e., $\chi_{\rm max} = 1600$, is enough to capture the essential physics of the ground state. Thus, we fix $\chi_{\rm max}$ to 1600 in all calculations. Although we did not present the results here, we however directly confirmed that keeping 1600 states provides convergent results in the finite system regardless of disorder.

As a benchmark result, we present in Fig. S3 the DMRG results of magnetization profile in the clean system. The system is at half-filling ($\delta N = 0$) with size $(L_x, L_y) = (30, 8)$, as shown in Fig. S3(a). Expectedly, the ground state shows the Néel order

where spins at each zigzag edge align antiparallel to each other. In Fig. S3(b), the system is slightly away from the half-filling, i.e., $\delta N = 12$ with size $(L_x, L_y) = (120, 4)$. The doping introduces a spin-density wave. Hence, the magnetization oscillates along the ribbon direction.

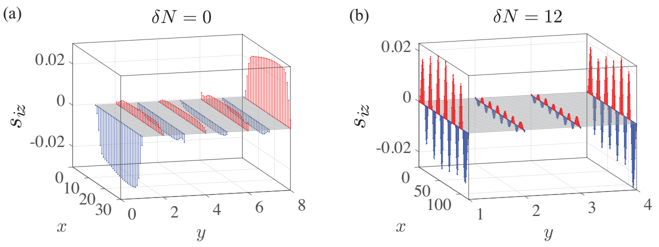

Fig. S3. Magnetization profile at (a) $(L_x, L_y, \delta N) = (30, 8, 0)$ and (b) $(L_x, L_y, \delta N) = (120, 4, 12)$ in the clean limit with $U/t = 1$.

II. HF APPROXIMATION AND TOPOLOGICAL ENTANGLEMENT SPECTRUM.

The ground state entanglement spectrum[2] is computed at half-filling using the HF approach. The spectrum differs from that of disorder-free SPT zigzag ribbons: degenerate eigenvalues of the SPT phase are split and distributed similarly to that of the DOS of the edge states of a disordered TO zigzag ribbon. The entanglement spectrum may be obtained from the reduced density matrix. To compute the bulk entanglement spectrum, we choose the region[3] A separate from the zigzag edges. We use the HF approximation; thus, the relevant reduced density matrix for a region A can be written as[4]

$$\rho_A = K e^{-\tilde{h}}. \tag{S12}$$

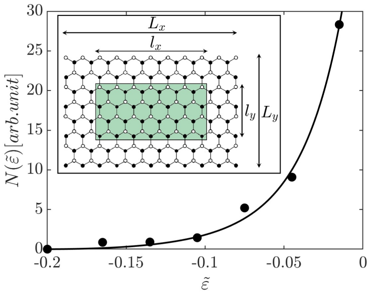

Fig. S4. Consider a rectangular region with length $l_x = 76$ and width $l_y = 36$. The rectangular region is inside a ribbon with length $L_x = 150$ and width $L_y = 72$. Distribution of eigenvalues $\tilde{\varepsilon}_k$ of the reduced density matrix of this region is plotted (there are also positive values of $\tilde{\varepsilon}_k$, but the distribution is identical). The distribution follows the exponential curve $B[e^{(\tilde{\varepsilon}-\tilde{\varepsilon}_0)^2/\delta^2} - 1]$ (black solid line). The parameters are $\Gamma = 0.3t$, $n_{imp} = 0.1$, and $U = 0.5t$ $(g = 0.19)$. The number of disorder realization is $N_D \sim 50$.

When the operator $\tilde{h}$ is diagonalized, we get the following Hamiltonian matrix

$$\tilde{h}_{ij} = \sum_k \psi_k^*(i)\psi_k(j)\tilde{\varepsilon}_k, \tag{S13}$$

where $\tilde{\varepsilon}_k$ and $\psi_k(j)$ are eigenvalues and eigenstates of the "Hamiltonian" $\tilde{h}$, respectively. Note that this particular density matrix describes a Fermi gas at temperature $k_B T = 1$. The reduced density matrix of either spin-up or spin-down electrons is equal to

$$\rho_{ij} = Tr(\rho c_i^\dagger c_j) = \sum_k \psi_k^*(i)\psi_k(j)\frac{1}{e^{\tilde{\varepsilon}_k} + 1}, \tag{S14}$$

where $c_i = \sum_k \psi_k(i) a_k$, and a_k is the electron destruction operator corresponding to the eigenstate $\psi_k(i)$. The distribution of the eigenvalues $\tilde{\varepsilon}_k$ of $\tilde{h}$ is called the entanglement spectrum. (Note that $\tilde{\varepsilon}_k$ are not the eigenenergies of the Hartree–Fock Hamiltonian.) The eigenvalues of a density matrix are given as

$$\lambda_k = \frac{1}{e^{\tilde{\varepsilon}_k} + 1}. \tag{S15}$$

The values $\tilde{\varepsilon}_k \approx 0$, corresponding to $\lambda_k \approx 1/2$, dominate the entanglement[5]. The entanglement spectrum of the SPT phase of a disorder-free zigzag nanoribbon exhibits numerous nearly degenerate eigenvalues, thereby reflecting the presence of nearly degenerate edge states. Fig. S4 shows the HF entanglement spectrum of a disordered interacting graphene zigzag ribbon at half-filling. This entanglement spectrum is different from that of the disorder-free SPT phase of zigzag ribbons. In the presence of disorder, the degenerate eigenvalues of the entanglement spectrum are split and exponentially distributed in a way similar to that of the DOS of edge states in TO zigzag ribbons, as shown in Fig. 1(b) in main article. In contrast, the DOS of disorder-free SPT phase of zigzag ribbons has a van Hove singularity, see Fig. 1 in main article.

---

* corresponding author: eyang812@gmail.com

[1] Schollwöck, U. The density-matrix renormalization group in the age of matrix product states. *Annals of physics* **326**, 96–192, DOI: 10.1016/j.aop.2010.09.012 (2011).

[2] Li, H. & Haldane, F. D. M. Entanglement spectrum as a generalization of entanglement entropy: Identification of topological order in non-abelian fractional quantum hall effect states. *Phys. Rev. Lett.* **101**, 010504, DOI: 10.1103/PhysRevLett.101.010504 (2008).

[3] Jiang, H.-C., Wang, Z. & Balents, L. Identifying topological order by entanglement entropy. *Nature Phys* **8**, 902–905, DOI: 10.1038/nphys2465 (2012).

[4] Peschel, I. Calculation of reduced density matrices from correlation functions. *Journal of Physics A: Mathematical and General* **36**, L205, DOI: 10.1088/0305-4470/36/14/101 (2003).

[5] Pouranvari, M. & Yang, K. Entanglement spectrum and entangled modes of random *xx* spin chains. *Phys. Rev. B* **88**, 075123, DOI: 10.1103/PhysRevB.88.075123 (2013).
